# Reasons for the Invasive Success of a Guppy (*Poecilia reticulata*) Population in Trinidad

**DOI:** 10.1371/journal.pone.0038404

**Published:** 2012-05-31

**Authors:** Caya Sievers, Eva-Maria Willing, Margarete Hoffmann, Christine Dreyer, Indar Ramnarine, Anne Magurran

**Affiliations:** 1 Scottish Oceans Institute, University of St. Andrews, St. Andrews, Fife, United Kingdom; 2 Department of Molecular Biology, Max Planck Institute for Developmental Biology, Tübingen, Germany; 3 Department of Life Sciences, University of the West Indies, St. Augustine, Trinidad and Tobago; University of Florida, United States of America

## Abstract

The introduction of non-native species into new habitats poses a major threat to native populations. Of particular interest, though often overlooked, are introductions of populations that are not fully reproductively isolated from native individuals and can hybridize with them. To address this important topic we used different approaches in a multi-pronged study, combining the effects of mate choice, shoaling behaviour and genetics. Here we present evidence that behavioural traits such as shoaling and mate choice can promote population mixing if individuals do not distinguish between native and foreign conspecifics. We examined this in the context of two guppy (*Poecilia reticulata*) populations that have been subject to an introduction and subsequent population mixing event in Trinidad. The introduction of Guanapo River guppies into the Turure River more than 50 years ago led to a marked reduction of the original genotype. In our experiments, female guppies did not distinguish between shoaling partners when given the choice between native and foreign individuals. Introduced fish are therefore likely to benefit from the protection of a shoal and will improve their survival chances as a result. The additional finding that male guppies do not discriminate between females on the basis of origin will further increase the process of population mixing, especially if males encounter mixed shoals. In a mesocosm experiment, in which the native and foreign populations were allowed to mate freely, we found, as expected on the basis of these behavioural interactions, that the distribution of offspring genotypes could be predicted from the proportions of the two types of founding fish. This result suggests that stochastic and environmental processes have reinforced the biological ones to bring about the genetic dominance of the invading population in the Turure River. Re-sampling the Turure for genetic analysis using SNP markers confirmed the population mixing process and showed that it is an on-going process in this river and has led to the nearly complete disappearance of the original genotype.

## Introduction

Invasive species now represent one of the greatest threats to biodiversity [1]. Humans have intentionally and unintentionally assisted species to invade new territories for thousands of years, but the dramatic increase of exotic species during the 20^th^ century and into the 21^st^ century is linked to growing international commerce and travel [2], [3]. Clavero and García-Berthou [4] point out that invasive species are the most important cause of bird extinctions and the second most important cause of fish and mammal extinctions worldwide. For instance, the release of the Nile perch (*Lates nilotica*) into Lake Victoria in the 1950s led to the extinction or extirpation of more than a hundred endemic cichlid species [5]. A feature of invaded communities is that they become more similar – biotically homogenized – in their species composition [6].

Of course, species diversity is only one facet of biodiversity; the concept includes genetic diversity as well [7]. Loss of genetic diversity is likely to be widespread, but is less comprehensively documented than the loss of species diversity. One context in which genetic diversity is threatened is when taxa that are not completely reproductively isolated come in contact with one another. For example, hybridisation between the North American mallard (*Anas platyrhynchos*) and the native New Zealand grey duck (*Anas superciliosa*) endangers the survival of the latter as a distinct species [8]. Intraspecific hybridisation can homogenise discrete characteristics of geographically isolated populations [9] and as a result can affect individual fitness by destroying local adaptations and adaptive variation [10]. One well-known example of introduced individuals endangering native stock of the same species is the escape and subsequent spawning of farmed Atlantic salmon (*Salmo salar*) with other Salmonids into the wild. Farmed salmon have been under artificial growth selection for decades and differ genetically from wild populations. In the wild, where they mix with native populations, their offspring experience high mortality rates which leads to concern for fitness and productivity of native populations due to large numbers of escaped fish [11]. Small populations are especially vulnerable to hybridisation in several respects including infertility of offspring, genetic homogenisation and outbreeding depression [12].

To understand, effectively control, and prevent invasions there is a need for long-term and large-scale strategies, as these are likely to be more successful and economical than trying to combat individual invaders [13]. Investigating the role of ecological, genetic and life-history characteristics of invasive organisms has therefore been a central focus of ecological research and experiments in the last five decades. It is only recently, however, that the role of behaviour during an invasion has received attention. Since behavioural adaptations can underpin successful invasion, this is a crucial theme for future research. In particular, it is vital to understand how behaviour influences the competitive ability and spread of invasive species [14]. The formation of large super-colonies by some invasive ants is an example of how behavioural shifts after introduction can increase the invasiveness of species [15]. In their natural habitat, the Argentine ant (*Linepithema humile*) exhibits strong intra-specific aggression. However, invasive populations in California almost completely lack this form of aggression. This leads to the formation of extremely dense and large colonies that are more competitive than native ant species in this region [16]. In a recent paper Cote et al. [17] showed that invasive mosquitofish (*Gambusis affinis*) have consistent individual differences in social behaviour, with asocial fish dispersing further than social ones. The authors suggest that this could have implications on the speed and impact of an invasion, should the invasive front consist of a biased subset of the population rather than a random group of individuals. Individual variation in behaviour can therefore influence the invasive success of a population [14].

There are multiple ways in which behaviour could contribution to invasiveness. Two types of behaviour that are likely to be particularly important are grouping behaviour and mate choice. Grouping behaviour, such as flocking in birds and shoaling in fish, provides protection against predation. Many species engage in such behaviours. For example it is estimated that around 50% of fish shoal at some point during their lives [18]. Factors such as the confusion effect and dilution effect mean that simply by joining a shoal or a flock, an individual will increase its chances of surviving a predator attack. Gregarious behaviour will thus promote contact between established and invading taxa, particularly if they are morphologically similar. In doing so it will enable small populations of colonising individuals to take advantage of the safety in numbers benefits of grouping. In addition these associations will facilitate mating between invading and native individuals. This is particularly likely to be important if mating partners do not discriminate against one another on the basis of genetic distance. Indeed, in many cases there is evidence of a preference for unusual or ‘rare’ mating partners, e.g. [19].

The Trinidadian guppy, *Poecilia reticulata*, a small freshwater fish native to Trinidad and Venezuela, is an ideal model system for testing the prediction that grouping behaviour will lead to mixing between native individuals and invaders. Guppy populations from the Caroni and Oropuche drainages belong to genetically distinct groups, with between 3.9 and 5.6% mtDNA variation [20] and show some, but incomplete, reproductive isolation [20], [21], but see [22]. Bringing fish from these different drainage systems into secondary contact could reverse the speciation process, lead to the loss of distinctiveness between rivers, genetic homogenisation and ultimately a loss of biodiversity. In fact this is already happening as a result of an experimental translocation of guppies over 50 years ago.

In 1957, C.P. Haskins transferred 200 guppies (approximately half of them female) from a high predation habitat in the lower Guanapo River (Caroni drainage) to the upper Turure River (Oropuche drainage) [23]. The place of introduction was previously guppy-free and contained only one other fish species, *Rivulus hartii*, a minor guppy predator [24]. A barrier waterfall isolated this part of the Turure from the lower parts of the river that were inhabited by a native guppy population.

In 1992, genetic investigations revealed that the newly arrived Guanapo fish had established a viable population, overcome the natural barrier of their habitat, perhaps with the help of flooding events that are common throughout the wet season, and started to spread below the waterfall, thereby coming in contact with the native Turure population. Fish from the upper and middle parts of the Turure displayed a high number of Guanapo alleles while fish from a downstream site of the Turure close to its confluence with the Quare still retained the native genotype [23]. In 2010, Willing et al. [25] examined the population history of guppies using SNPs and affirmed that fish from the middle stretches of the Turure clustered with populations from the Caroni drainage rather than the Oropuche drainage.

Here we test the hypothesis that behaviour plays a role in integrating invading and native guppies. We first examine the mechanisms that enable invading fish to gain a foothold in a new environment. Specifically we test the prediction that the strong shoaling tendency in this species means that fish that are morphologically similar will tend to associate with one another, irrespective of their origin. Because females are core members of shoals [26], and devote a larger fraction of their time to antipredator behaviour, we focus on their choices. We further predict that male guppies will not discriminate between native and foreign females in a mating context. Although male guppies exhibit preferences based on female size, reproductive status [27] and familiarity [28], they also engage in persistent courtship and will even attempt matings with females belonging to different poeciliid species [29] and families (goodeids, e.g. *Skiffia bilineata*) [30]. We use fish from the original source site in the Guanapo River and individuals from the Oropuche, a river in close proximity to the Turure that is inhabited by fish that are genetically similar to the ancestral (pre-invasion) population.

In the second part of the paper we test the prediction that the behavioural integration of native and invading fish, mediated by the shoaling and mating choices identified above, will lead to interbreeding with the mating success of the two forms proportional to their abundance. The alternative hypothesis is that Caroni (invading) males may outcompete indigenous Oropuche males during mating [31]. This is supported by the observation that the genotypes of fish in the invaded section of the Turure River strongly resemble those in the Caroni drainage system. To distinguish between these possibilities we conducted a mesocosm experiment in which we introduce fish from the Guanapo and Oropuche in different proportions and leave them to interact freely. After one year, fish from all mesocosms were counted and 30 juveniles taken for genetic analysis.

Finally, to examine the current state of the Turure population and the geographic extent of the invasion of Guanapo fish, we re-sampled several sites along this river, as well as the site of Haskins's source at the Guanapo and two sites at the Oropuche. Here we test the prediction that Guanapo fish will have continued to spread downstream since the last investigation and further reduced the natural Oropuche genotype.

Single nucleotide polymorphism (SNP) markers specific for Guanapo and Oropuche (Turure) fish were used to examine the current genetic composure of populations from different sites along the Turure and the mesocosm experiments. In most cases, SNPs markers are bi-allelic. An advantage for genetic mapping and sequencing is the high abundance with which SNPs occur throughout the entire genome [32]. In contrast to microsatellites that frequently have multiple alleles, it is easy to calculate the proportions of alleles from each parental population based on the two alleles per SNP. SNPs are therefore very useful when analysing closely related populations/species.

## Methods

All experiments were carried out between 2008 and 2010 at the University of West Indies in Trinidad, using wild caught guppies from the Guanapo River (Caroni drainage, Haskins' source) and Oropuche River (Oropuche Drainage, Valencia Road). For behavioural experiments, fish were transferred to stock tanks and kept on a 12 L∶12 D cycle using 18 W fluorescent daylight bulbs. The tank temperature was maintained at 26±0.5°C. Aged tab water was used to fill the tanks. The water in newly set-up tanks was treated with STRESS COAT®. Fish were fed with TetraMin flake food in the morning about half an hour before the first trial and freshly hatched Artemia larvae in the evening. Stock tanks contained a filter, a plastic plant and some *Elodea canadensis* for cover. Black plastic was used to form a visual barrier the tanks. No permit was needed for any of the experiments described here.

### Behavioural experiments

#### Female shoaling behaviour: do females discriminate between native and foreign shoal mates?

After capture, fish were given five to six days to acclimatise to laboratory conditions before trials started. Trials for this experiment took place in April 2009. Experimental females were split into three groups per population, and each group transferred into its own tank (45×31×30 cm; approximately 20 fish per tank), to prevent them from becoming familiar with other experimental fish. The trials took place between 0800 hours and 1700 hours using two identical choice tanks (45×31×30 cm) with a gravel bottom and filled with water to 20 cm. Three sides of each tank were covered in black plastic to provide a uniform background. Two clear 600 ml Plexiglas bottles (∅7 cm) containing several small holes for water exchange were placed at either end of the tank. Each bottle was stocked with 3 size matched females from the Guanapo or Oropuche, respectively, all of them unfamiliar with the focal fish. One set of companion females was typically used for three or four trials and afterwards transferred back into their stock tank. Before each trial the bottles containing the fish were randomly placed at both ends of the experimental tank, where the fish quickly settled. All companion females displayed normal swimming and shoaling behaviour. The focal female was carefully transferred into a small ‘start box’ in the middle of the back site of the tank. The trial started as soon as this fish left the box and swam freely around the tank. For 10 min. the time the female spent within 6 cm (approximately 2 body length) of both bottles was recorded. 26 Guanapo females and 28 fish from the Oropuche were tested. Female total length ( = from tip of snout to end of caudal fin) was measured after the trial before they were transferred back into their home tank.

#### Male choice behaviour: do males distinguish between native and foreign mating partners?

After capture, fish were transferred into six stock tanks (approximately 20 fish per tank) and given five days to acclimatise to laboratory conditions before trials started. Trials for this experiment took place in May 2008. All trials took place between 0800 hours and 1600 hours in a choice tank (60×30×20 cm deep) similar to the one described before. However, instead of using clear plastic bottles to hold female groups, the tank was divided into 3 compartments by clear perforated Plexiglas that allowed water circulation. Both side compartments measured 9 cm in length while the middle compartment measured 42 cm. Two 6 cm long choice zones were marked with thin black lines in front of the side compartments; a distance that equals approximately three body lengths in adult male guppies. This tank set-up has commonly been used in choice experiments testing for female choice in guppies, e.g. [33] & [34]. The two end compartments of the observation tank were stocked with three size matched Oropuche or three Guanapo females, respectively. Fish were allowed at least one hour to settle down. Before the trial, a focal male was gently placed into a clear plastic bottle in the middle compartment of the observation tank and allowed 5 min. to settle before the bottle was carefully removed. The time the focal male spent in each choice zone was then measured and the number of sigmoid displays, a form of courtship behaviour, towards females was recorded for 15 min. At the end of each trial, the total length of the male was measured. Then he was transferred back into one of the population stock tanks and played no further part in this experiment. A total of 46 males (23 per population) were tested.

### Mesocosm experiment: does behaviour promote population mixing?

Twenty-four mesocosm tanks were set up in a fenced compound at UWI campus in St Augustine, Trinidad in December 2008. Each of them had a diameter of 1.2 m and height of 80 cm. An overflow in the form of a short tube covered with a fine mesh at the inner end to hold water levels constant and prevent fish from being washed out during rainfall, was fitted to each mesocosm at a height of 60 cm. A lid made out of mesh wire on top of all mesocosms was used to stop dead leaves from falling into the water as well as to avoid any bird predation on guppies. Tanks contained small and medium sized stones for cover, especially for newborn fish, and some plants to provide extra shade.

This experiment consisted of five different treatments, each replicated three times (100% Oropuche; 100% Guanapo) or six times (Oropuche 20%–80% Guanapo, 50%-50%, 80%-20%), respectively. Treatments were randomly assigned to mesocosms across the compound. Each tank was then stocked with 20 wild adult guppies from the Oropuche and/or Guanapo according to the treatments. The same number of males and females per population were used, e.g. a mesocosm containing an Oropuche 20∶80 Guanapo treatment would be stocked with two Oropuche males and females and eight Guanapo males and females each. After their transfer to the mesocosms, fish were not fed, but lived on natural productivity (algae and small freshwater invertebrates). Several large trees and hedges on both sides of the compound provided shade or half-shade throughout the day. In January and February 2010, after 13 to 14 months and approximately 4–5 guppy generations, all fish in each mesocosm were caught using a hand net. 30 juveniles per mesocosm were randomly chosen and killed with an overdose of Tricaine Methanesulfonate, fixed in 96% ethanol and stored at −20°C. Juveniles were used to maximise the chances of detecting population mixing that had taken place since the start of the experiment. All remaining fish were released in an isolated artificial pond at UWI campus.

In June and July 2010, all fish samples were taken to the Max Planck Institute for Developmental Biology in Tuebingen, Germany, for genetic analysis. Here population specific markers already existed due to a genome-wide SNP study of guppy population history in Trinidad and Venezuela [25].

2 to 3 mg of dried body tissue (avoiding the gut) per newborn was transferred into a 1.5 ml microcentrifuge tube. DNA was isolated using the DNeasy Blood & Tissue Kit from Qiagen. The tissue of five newborns was pooled in one tube, leading to six tubes per mesocosm, each containing around 10 to 15 mg of tissue and the DNA adjusted to an end-concentration of 25 ng/µl.

For DNA amplification the following PCR protocol was used: 2.5 µl [25 ng/µl] template, 4.4 µl H_2_O, 1 µl PCR buffer [10× Thermobuffer (NEB B9004S)], 1 µl dNTPs [2 mM], 0.1 µl Taq polymerase [5000 U/m (NEB M0267L)], 0.5 µl Fw-primer and 0.5 µl Rev-primer [both 10 pmol/µl]. All PCR amplifications run in Peltier Thermal Cyclers pTC-200 from Bio-Rad under the following conditions: 96°C for 3 min, 40 cycles of 96°C for 30 sec., 56°C for 30 sec., 68°C for 30 sec.; 68°C for 5 min.

The markers used in this experiment were developed by Tripathi et al. [35] for a complete linkage map of the guppy. Before genetic analysis, 28 markers were selected that were likely to distinguish between Guanapo and Oropuche (Turure) populations, based on the study on guppy population history by Willing et al. [25]. Out of these, 4 produced clear results in pure Guanapo and Oropuche fish, therefore only these four markers were used in this experiment. Four different loci were represented by the chosen markers (Table 1).

**Table 1:** Primer sequences.

Marker	Forward primer	Reverse primer	Linkage group	GenBank accession no	Number of SNPs
M 654	TTTACATCCCACACCTTCAATC	TGTGAATGCTCAACCAAACTC	LG02	FH890962	8
M 978	GGCCCATCTGGATAGAGTG	TTAACATCTTGTGGAGTTATGCTG	LG23	FH893187	5
M 1033	AATCAGTCAGTTTACAAAGTCTGGTC	TGGAGACGCAATCAGTGG	LG10	FH893635	1
M 1042	ACAACATTCTATGGGTGAAAGAAG	GCTCATTGTAAGGGTAGTGTGC	LG09	FI903158	1

Sequences of forward and reverse primer for each marker that produced clear results in pure Guanapo and Oropuche fish. The linkage group of all markers and the number of resulting SNPs per marker are shown. (Sequences from: [31], Data supplement no 2 & 3).

After the removal of excessive primers (using 1.2 µl Exo I E.coli and 1.2 µl FastAP), the mix was prepared for sequencing: 1 µl template [25 ng/µl], 6 µl H_2_O, 2 µl Sequencing buffer [5× Sequencing buffer (AB 4305603), 0.5 µl Fw-primer [10 pmol/µl] and 0.5 µl BDT [Big Dye Terminator v3.1 from Applied Biosystems Cat. no. 4336921 (undiluted)]. The following sequencing program was used for amplification: 96°C for 30 sec., 40 cycles of 96°C for 20 sec., 50°C for 10 sec. and 60°C for 4 min. The amplified product was then sequenced, using a sequencer from Applied Biosystems ABI 3730 XL. The final DNA sequences for each marker were analysed with the help of the pregap4 and gap4 software from the Staden-package (GAP v 4.10) to investigate all SNPs resulting from the four markers that were informative between the Guanapo and Turure (Oropuche) populations. The percentage of Oropuche nucleotides compared to Guanapo nucleotides was determined for all SNPs per marker by estimating the proportion of the area of both peaks of the consensus sequence in per cent in case two nucleotides were present.

The mean percentage of all Oropuche allele frequencies estimated at four different loci with 14 SNPs per treatment was then used to investigate if these resembled the proportion of founding fish or if one population contributed significantly more to the observed allele frequencies in the offspring. Results were bootstrapped (n = 1000) to obtain 95% CI. To account for the markers not being entirely homozygous for both populations, expected values were calculated based on the observed allele frequencies in the two pure populations (Oropuche 100% and Guanapo 100%). Observed proportions of pure Oropuche and Guanapo treatments (ranging from 0 to 1.0) were combined into 20 bins with an interval size of 0.05, respectively. These bins were then used in a bootstrapping approach (n = 1000) where a single value was sampled from the pool of results to obtain probability distributions for both treatments. Based on these probability distributions, the expected values were calculated taking the proportion of founding fish from both populations into account (e.g. 24 values were drawn from the probability distribution of pure Oropuche fish and 6 values from the distribution of pure Guanapo fish, respectively, to obtain an expected mean value for the 30 analysed juveniles from the Guanapo 20–Oropuche 80 treatment). Wilcoxon signed rank tests were then carried out for the mixed treatments to test for differences between the observed amount of Oropuche nucleotides found in 14 SNPs and the values expected in accordance with the initial proportion of founding fish from both populations.

### Re-sampling of the Turure: is the invasive genotype continuing to spread through the system?

Fish used for the genetic analysis were caught at eight sites across the Caroni and Oropuche drainages the Northern Mountain Range, Trinidad, using a seine net and hand nets. Ten adult fish per site (both males and females) were killed with an overdose of Tricaine Methanesulfonate immediately after catching, transferred into 96% Ethanol and stored in a freezer. Only three adults could be caught at the Toco Main Road site of the Oropuche. The grid references of all sites are listed in Table 2.

**Table 2:** Grid references of catching sites.

Population	Grid reference number	Site name	Drainage system
Guanapo	PS 913 765	Eastern Main Road (Haskins's source)	Caroni
Oropuche	QS 042 788	Valencia Road	Oropuche
Oropuche	QS 078 719	Toco Main Road	Oropuche
Turure	PS 997 817	Haskins's introduction	Oropuche
Turure	PS 997 817	Below waterfall	Oropuche
Turure	QS 003 809	Cumaca Bridge	Oropuche
Turure	QS 002 784	Valencia Road	Oropuche
Turure	QS 023 738	Confluence with Quare	Oropuche

Grid references of sites used to catch fish for the re-sampling of the Turure, behavioural and mesocosm experiments. The drainage system each river belongs to is indicated.

**Figure 1:**
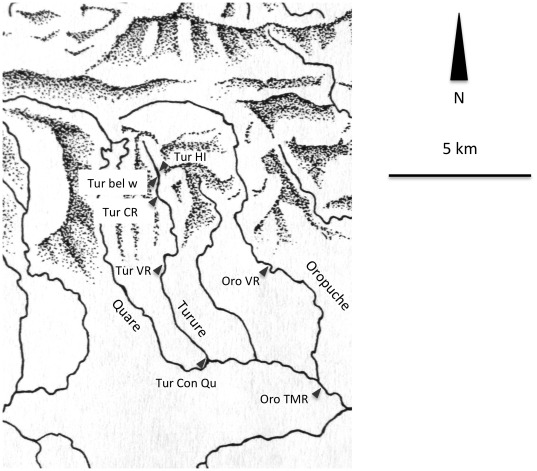
Collection sites at the Northern Mountain Range. Detail of the Northern Mountain Range map showing the Turure and Oropuche Rivers. Red arrows indicate the sites where fish collection took place. HI: Haskins's introduction, bel w: below waterfall at introduction site, CB: Cumaca Bridge, VR: Valencia Road, Con Qu: Confluence with Quare, TMR: Toco Main Road. Not shown: Haskins's source, Guanapo.

Sampling took place in June 2009, except for the Haskins Introduction site and directly below the nearby waterfall (both Turure), where no fish were present at that time. Fish from these two sites were collected in June 2010 (see Figure 1 for a map showing all collection sites).

DNA was isolated using the DNeasy Blood & Tissue Kit from Qiagen. The tissue of five adult fish was pooled in one reaction tube. After isolation, the two samples per site were combined, leading to a pool of ten fish per sample. The rest of the protocol followed the steps already described above.

The percentage of the original Turure allele frequencies estimated at four different loci with 14 SNPs compared to the introduced Guanapo genotype was determined for each SNP in the same way as for the mesocosm experiment. The mean results for all SNPs per marker per site were then used to investigate the genetic change that took place in the Turure after the introduction of Guanapo fish and the following invasion of the middle and lower parts of the river.

## Results

### Behavioural experiments

#### Female shoaling behaviour: do females discriminate between native and foreign shoal mates?

**Figure 2:**
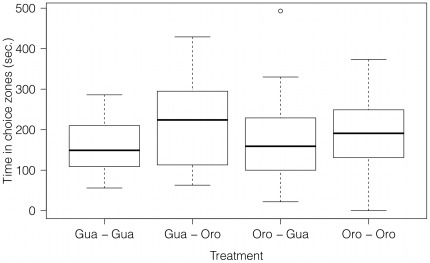
Shoaling times of Guanapo and Oropuche females. The amount of time focal females from either the Guanapo or Oropuche spent shoaling with fish from their own or the other population. Medians and interquartile ranges are shown.

Guanapo and Oropuche females used in the 2009 shoaling experiment were of equal size (t-test, t = −0.98, d.f. = 53, P = 0.332). A significant difference between the total time females of both populations spent shoaling was found (One-way ANOVA, F = 7.22, d.f. = 1,218, P = 0.008). Guanapo females on average shoaled for 363.3 sec±116.1 S.D. out of 600 sec whereas Oropuche fish only spent a mean time of 327.4 sec±80.9 S.D. close to their companion fish (Figure 2). To test if a female spent more time with her own population than with the other population, the time she spent shoaling with the other population was subtracted from the time she spent with her own population. These values were then tested for a significant difference from 0. Guanapo fish did not distinguish between shoaling partners from their own population and Oropuche females (One-sample t test, t = −1.79, d.f. = 25, P = 0.086). Similarly, Oropuche females showed no preference for shoaling with individuals from their own population over shoaling with Guanapo fish (One-sample t test, t = 0.53, d.f. = 28, P = 0.597).

#### Male choice behaviour: do males distinguish between native and foreign mating partners?

**Figure 3:**
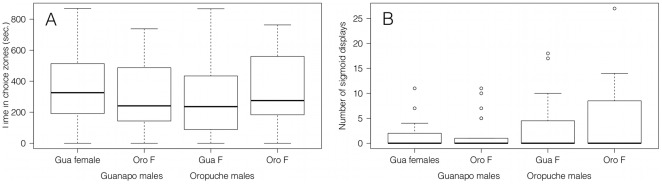
Male mate choice. The time focal males from the Guanapo and Oropuche spent in the choice zones with a set of females from either their own or the other population (a) and the number of sigmoid displays they directed towards these females (b). Medians interquartile ranges and outliers are shown.

No size difference existed between males (Mann-Whitney U test, Z = −1.829, n_Gua_ = 23, n_Oro_ = 23, P = 0.067) or females (Mann-Whitney U test, Z = −0.895, n_Gua_ = 12, n_Oro_ = 12, P = 0.371) from the Guanapo and Oropuche used for the male mate choice trials. Guanapo males had a mean size of 2.01±0.13 cm, while Oropuche males were on average 2.09±0.13 cm. Guanapo females had a mean size of 2.33±0.21 cm, and Oropuche females had an average size of 2.47±0.35 cm. No difference was found between the total time males from the Guanapo or Oropuche spent in the two choice zones (Mann-Whitney U test, Z = −0.692, n_Gua_ = 23, n_Oro_ = 23, P = 0.489). Guanapo males did not spend significantly more time in the Guanapo choice zone than did Oropuche fish (Mann-Whitney U test, Z = −1.066, n_Gua_ = 23, n_Oro_ = 23, P = 0.286). Likewise, Oropuche males did not spend more time in the Oropuche choice zone with females from their own population than did Guanapo males (Mann-Whitney U test, Z = −0.781, n_Gua_ = 23, n_Oro_ = 23, P = 0.435) (Figure 3a).

The total number of sigmoid displays directed towards females did not differ between Guanapo and Oropuche males (Mann-Whitney U test, Z = −1.304, n_Gua_ = 23, n_Oro_ = 23, P = 0.192). Guanapo males did not display more often towards Guanapo females than did Oropuche males (Mann-Whitney U test, Z = −0.087, n_Gua_ = 23, n_Oro_ = 23, P = 0.930). Similarly, Oropuche males did not direct more sigmoid displays towards Oropuche females than did Guanapo males (Mann-Whitney U test, Z = −0.969, n_Gua_ = 23, n_Oro_ = 23, P = 0.332) (Figure 3b).

### Mesocosm experiment: does behaviour promote population mixing?

**Figure 4:**
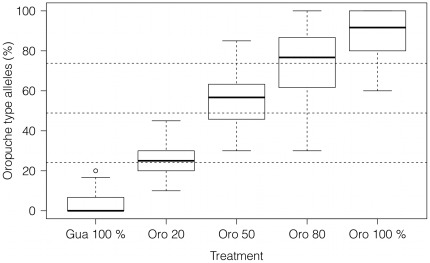
Percentage of SNPs from the mesocosm experiment. The percentage of Oropuche nucleotides scored at 14 SNPs across different four loci that distinguished between Guanapo and Oropuche origin for all treatments. Dashed lines at 20, 50 and 80% indicate where a theoretical mean for each treatment would be expected given that only the proportion of introduced fish is important for the distribution of Guanapo and Oropuche allele frequencies. Medians, interquartile ranges and outliers are shown.

The mean number of all fish per mesocosm did not differ between treatments (Kruskal-Wallis test, χ^2^ = 6.24, d.f. = 4, P = 0.182). Thirty newborns per mesocosm were used for genetic analysis. This corresponded with a mean of 22.5%±19.9 S.D. of total offspring per mesocosm, ranging from 4.4% to, in one case, 100% (Mesocosm no 2, Guanapo 20∶80 Oropuche). The four markers used in the genetic analysis of the re-sampled Turure fish resulted in 14 meaningful SNPs across the four loci that could be analysed. These SNPs distinguished clearly between Guanapo (Caroni) and Turure (Oropuche) origin. Nucleotide frequencies are reported as per cent of Oropuche nucleotides found in each treatment (Figure 4). The SNPs used were not entirely homozygous for both populations (Guanapo mean = 3.97%; Oropuche mean = 89.84%). However, a clear trend is visible in all treatments, and the allele frequencies at the four loci reflect the proportions of initially introduced fish. Observed allele frequencies in fish belonging to the Guanapo 80∶20 Oropuche did not significantly differ from the value expected (Mean = 25.43% vs. the expected 24.10%, Wilcoxon signed rank test, p = 0.191), nor did allele frequencies from the Guanapo 20∶80 Oropuche samples (Mean = 73.76% vs. 73.80%, Wilcoxon signed rank test, p = 0.662). Allele frequencies at the four different loci within the sample the Guanapo 50∶50 Oropuche treatment showed a slight excess of Oropuche nucleotides (Mean = 55.99% vs. 48.90%, Wilcoxon signed rank test, p<0.001). See Table 3 for bootstrap results, 95% CI, expected values and results of Wilcoxon signed rank tests.

**Table 3:** Proportion and test results of SNPs in the mesocosm experiment.

Treatment	Mean (%)	CI (0.025)	CI (0.975)	Expected mean (%)	P-value
Guanapo 100%	3.97	2.38	5.87	-	-
Gua 80∶20 Oro	25.43	23.83	26.85	24.10	0.191
Gua 50∶50 Oro	55.99	53.03	58.85	48.90	<0.001
Gua 20∶80 Oro	73.76	69.43	77.68	73.80	0.662
Oropuche 100%	89.84	86.43	92.97	-	-

Results of Wilcoxon signed rank tests that compared the actual mean of Oropuche allele frequencies estimated at four different loci with 14 SNPs (in per cent) to a theoretical value that would have been expected according to the initial proportions of Guanapo and Oropuche fish when the error due to not completely homozygous markers is taken into account. 95% CI were obtained by bootstrapping (n = 1000).

### Re-sampling of the Turure: is the invasive genotype continuing to spread through the system?

**Figure 5:**
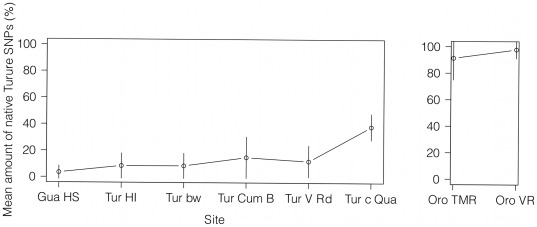
Percentage of SNPs from re-sampling the Turure river. The amount of native Turure and Oropuche allele frequencies estimated at four different loci with 14 SNPs compared to the invading Guanapo allele frequencies. The percentage of the Oropuche state of all SNPs per site was taken in order to illustrate the change of genetic composition across the river Turure. Two sites at the Oropuche were sampled to compare the Turure results to a likely original genotype. N = 10 fish per site, except Oropuche Toco Main Road: n = 3. HS: Haskins's source; HI: Haskins's introduction. Error bars represent 95% CI.

The four markers used in the genetic analysis of the re-sampled Turure fish resulted in 14 meaningful SNPs across the four loci. As can be seen in Figure 5, allele frequencies at the four different loci represented by 14 SNPs from population samples collected at Haskins's source at the Guanapo differ remarkably from the ones typically found in populations from the Oropuche and are nearly homozygous (Mean amount of SNPs identical with the nucleotide typically found in Oropuche fish: 3.57%±4.97 S.D.). The allele frequency displaying the original Oropuche (Turure) state is slightly higher at the introduction site in the Turure and directly below the barrier waterfall (both sites: 8.57%±9.49 S.D.). One kilometre further downstream, at Cumaca Bridge, the percentage of alleles displaying the native state has doubled (15.00%±15.57 S.D.) and nearly is the same in Turure fish caught at Valencia Road (12.14%±11.88 S.D.). At the confluence of Turure and Quare, nearly 40% of tested alleles at the four different loci displayed the original Turure state (38.18%±9.82 S.D.). In comparison, 98% of Oropuche fish collected at Valencia Road displayed an Oropuche typical nucleotide at each SNP (97.78%±6.67 S.D.). This value was slightly lower at Toco Main Road (91.25%, ±16.42 S.D.), but only three fish could be used for genetic analysis at this site (Figure 5).

## Discussion

Female guppies of the Oropuche and Guanapo did not distinguish between shoaling partners and spent the same amount of time with females from their own and the other population in a choice experiment. Similarly, male guppies did not discriminate between female origin, and courted females independent of population. These results confirm that shoaling behaviour (by females) and mating behaviour (by males) have the potential to promote integration of native and foreign fish. When populations were left to mate freely in a long-term mesocosm experiment, invasive Guanapo guppies did not have an intrinsic reproductive advantage over Oropuche fish as was previously thought. The allele frequencies at four different loci with 14 SNPs estimated within samples were consistent with the values expected given the proportion of founding fish, once the homozygosity of the markers was taken into account. Slight variation of these values might be due stochastic effects after only four or five generations of population mixing. As expected, the re-sampling of the Turure confirmed the hypothesis that Guanapo fish continued to expand downstream, with alleles of the invasive population found in the entire river from the introduction site to its confluence with the Quare 10 km further downstream.

Intra-specific hybridisation can quickly homogenise the unique characteristics of geographically distinct populations as well as influence the fitness of individuals by disrupting local adaptations [36] and behaviour can play an important role in the speed and direction of population mixing. With or without introgression, hybridisation between species or genetically distinct populations often endangers their survival in a wide variety of plant and animal taxa [37]. When Hindar et al. [11] simulated the future of wild Atlantic salmon (*Salmo salar*) populations using different intrusion rates of escaped farmed animals, the authors found that even with a medium intrusion rate of 20% extensive changes in the population structure of wild salmon would occur within around 10 generations. The analysis of allozymes and mtDNA in an endemic pupfish species (*Cyprinodon bovinus*) in Texas revealed that all wild populations of this fish contain foreign genetic elements (ranging from 6.1 to 15.1%) from the sheepshead minnow (*Cyprinodon variegatus*), a recently introduced species. The last individuals with the original, pure *C bovinus* genotype that do not contain genes from the sheepshead minnow belong to a laboratory population [38]. Another example of a high rate of hybridisation is reported by Hänfling et al. [39], examining the native British crucian carp (*Carassius carassius*). Only 62% of tested populations consisted of the genetically pure native species. In all other populations, hybrids both with the goldfish (*C. auratus*) und the common carp (*Cyprinus carpio*) were common, sometimes to the extent that no pure crucian carps could be found. Again, this level of hybridisation endangered the genetic integrity and local adaptations of the native species.

Here females guppies did not distinguish between the population origin of their shoaling partners, but spent an equal amount of time with fish belonging to their own population and females of a different population. Magurran et al. [40] found that females from two populations preferred to shoal with familiar over unfamiliar fish from their own population, but did not discriminate between unfamiliar fish of their own and a different population. In their experiments females could only use visual cues to choose their shoaling partners, and had no access to odour cues. The availability of odour cues can be important, for example in differentiating between members of other groups or populations in the stickleback (*Gaterosteus aculeatus*). Here fish were able to discriminate between their own group and members of a different group originating just 200 metres away. This choice was based on the existence of habitat cues such as the differences in smell produced by feeding on different food sources [41]. In a later experiment, Ward et al. [42] found that guppies were able to distinguish between their own tank members and members from another tank based on the smell produced by different food and water sources. In the experiment described here, fish had access to visual as well as odour cues when choosing between shoaling partners. The inability to differentiate between their own and other populations could be explained by the same living and feeding conditions these fish experienced before and during the experiment, a situation similar to an invasive event. In an invasive event, as happened in the Turure, different populations would experience the same living conditions and exploit the same food resources, so that they are very likely to produce similar olfactory cues. It is therefore likely that mixed shoals were commonplace after the invasion took place. Despite not being able to distinguish between the population origin of shoaling partners, the occurrence of shoaling behaviour will increase the survival rate of shoal members, regardless of being native or introduced. This means shoaling with other fish has the potential to increase the speed with which introduced individuals spread and colonise their new habitat.

In the Turure, these shoals of mixed population origin will be encountered by male guppies on their search for mating partners. Unlike females, who were reported to slightly prefer mating with males from their own population [43], but see [31], male guppies did not discriminate between female origin but spent the same amount of time with females of both populations in the choice experiment presented here. This result was the same for males from the two different populations, the Guanapo and the Oropuche. The absence of a preference for females of their own population could either be due to the inability of males to distinguish between female origin or a general lack of male interest in the source of mating partners. However, the observed inability or unwillingness of males to differentiate between female origin can still influence the outcome of an invasive event and the speed of gene-pool mixing, even in the absence of obvious differences between the mating behaviour of male guppies from the Guanapo and the Oropuche. Because male guppies can overrule female choice with forced copulations that lead to sperm transfer [44], the role of female preference in ensuring that a female mates with males from her own population is weakened. It is therefore possible for populations to mix quickly after an invasive event without the invading males necessarily showing superior or more dominant mating behaviour than males belonging to the native population.

The genetic results obtained of the mesocosm experiments add another layer in understanding the outcome of Haskins's introduction. In an experiment looking at female mate choice and male mating success between fish belonging to the Tacarigua River (Caroni drainage) and the Oropuche (Oropuche drainage), Magurran et al. [31] found that while females did not prefer males from either population in a choice test, Tacarigua males secured all copulations in a mating experiment. The authors proposed that this dominance of Caroni type fish in achieving reproductive success could be an explanation for the success of Guanapo fish (Caroni drainage) over Turure fish (Oropuche drainage). In contrast to these results, we were not able to find any behavioural differences in the mating behaviour of males belonging to the Guanapo or the Oropuche. Guanapo allele frequencies in the analysed loci reflected the proportion of fish with which mesocosms were stocked originally. This result makes a general superiority of Guanapo males unlikely. In a study testing for male dominance behaviour, we could not find a difference in either dominance behaviour, frequency of courtship behaviour or mating success between Oropuche and Guanapo males (unpublished data). At the same time no differences in female fecundity existed between pure populations or mixed treatments. The number of offspring or the time until females gave birth did not differ in pure Oropuche and Guanapo or mixed treatments (Guanapo ♀ – Oropuche ♂ and vice versa) (unpublished results).

Beside an obviously much longer time scale and greater population size of guppies in the Turure, another factor distinguished the mesocosm experiment and Haskins's introduction. Our mesocosms consisted of isolated compartments from which fish were unable to leave and into which no individuals from outside populations could colonise. Accordingly there was no possibility for movement between populations and no corresponding gene flow. The constant influx of Guanapo fish from above the barrier waterfall into the middle and lower parts of the Turure and the subsequent gene flow and population mixing over a sufficient amount of time, combined with stochastic environmental events such as periods of flooding or pollution in the downstream section of the Turure, might be another explanation for the excess of Caroni genes today, despite the lack of behavioural differences or genetic superiority of the invaders. Future experiments could test for behavioural differences such as movement or predator evasion, or look at other factors that might affect relative survival and differ between both populations.

When comparing our results of re-sampling the Turure to the study published by Shaw et al. [23], it becomes apparent that in the 20 years following the first discovery of the introduction, Guanapo fish spread further downstream the Turure and displaced the native population and by today have moved beyond the Turure's confluence with the Quare. It also implies that both populations have not reached a stable equilibrium point as was suggested by Becher & Magurran [45], but that an introduction experiment carried out to better understand the ability of guppies to quickly adapt to new environments may ultimately end in the disappearance of an entire series of populations in one drainage system that were genetically distinct from guppy populations in other drainages. Allendorf & Leary [46] investigated the genetic integrity of the North American cutthroat trout (*Salmo clarki*) that consists of several genetically distinct subspecies, and found that a high percentage of examined populations showed evidence of hybridisation with rainbow trout (*Salmo gairdneri*) and/or released cutthroat trouts from hatcheries. Because introduced fish are often released in headwater lakes, they also threaten to genetically change downstream populations, thereby destroying local adaptations throughout a drainage. This development not only endangers the survival of the native Turure population which is now nearly extinct, but also the genetic diversity displayed by populations belonging to the Caroni and Oropuche drainage system. Despite this distinctiveness, populations show similar adaptations to predators and environmental gradients across streams. Trinidadian guppy populations can therefore be seen as series of naturally repeated experiments that give an insight into the working mechanisms of evolution [47].

Haskins's introduction is an example of how different types of behaviour can combine to influence the speed and direction of an invasion as well as gene pool mixing of not fully reproductively separated populations. While shoaling behaviour increases the chances of survival for all shoal members, it helps the non-natives to more successfully colonise a new territory. At the same time gene pools mix quickly due to the lacking preference of males for mating with females of their own population. No reproductive advantage existed for Guanapo guppies when left to mate freely, neither did pre- or post-zygotic barriers between populations prevent gene pool mixing after the introduction. This means that as soon as the first introduced individuals encountered the native Turure population, a speedy and invisible colonisation as well as population mixing could take place without a possibility to stop or reverse this ongoing event.

In the long run, the extirpation and possible extinction of the native population as well as the threat of an ongoing mixing of the invaders with populations living in rivers close to the Turure, is another example of biotic homogenisation. As seen in studies concentrating on other species (e.g. Atlantic salmon [11], New Zealand grey duck [8]), the contact between genetically distinct populations or closely related species after an introduction can easily destroy genetic variation and blur the borders between separated populations or species. In the long term, and keeping the many invasive events worldwide in mind, population mixing and genetic homogenisation will lead to the creation of a more uniform and impoverished form of biodiversity [36]. In the case of Haskins's introduction, this does not so much mean the replacement of a local specialist by a cosmopolitan generalist but rather the loss of a diverse range of different genotypes while a single population becomes more abundant. It is unlikely that this homogenisation of gene pools will endanger the survival of guppies as a species, but some of the diversity guppies became famous for in the first place will be lost as an unintended consequence of a scientific project devised over 50 years ago to understand exactly this diversity. Introduction experiments in Trinidad and elsewhere in the world are ongoing. With the knowledge of possible consequences of this sort of experiments, some of them shown in this study, great care should be taken with any kind of species translocation and alternative solutions such as mesocosm experiments and modelling should be considered.
